# Ethacrynic Acid Enhances the Antitumor Effects of Afatinib in EGFR/T790M-Mutated NSCLC by Inhibiting WNT/Beta-Catenin Pathway Activation

**DOI:** 10.1155/2021/5530673

**Published:** 2021-04-27

**Authors:** Xuehui Zhang, Chaoyuan Huang, Biyu Cui, Yebin Pang, Rong Liang, Xiaoling Luo

**Affiliations:** ^1^Department of Nuclear Medicine, The Ninth Affiliated Hospital of Guangxi Medical University, Beihai People's Hospital, Beihai, Guangxi, China; ^2^Department of Oncology, The Third Affiliated Hospital of Guangxi Medical University, Nanning, Guangxi, China; ^3^Department of Endocrinology, The Affiliated LiuTie Central Hospital of Guangxi Medical University, Liuzhou, China; ^4^Department of Gynecological Oncology, GuangXi Medical University Cancer Hospital, Nanning, Guangxi, China; ^5^Department of Digestive Oncology, GuangXi Medical University Cancer Hospital, Nanning, Guangxi, China; ^6^Research Department, GuangXi Medical University Cancer Hospital, Nanning, Guangxi, China

## Abstract

*Background*. Despite afatinib as a new first-line treatment for EGFR L858R and exon 19 deletion or other rare EGFR-mutation patients, the acquired resistance or toxic effects associated with it limited its use clinically. The controlling of acquired resistance or optimization of the afatinib dosage in EGFR/T790M mutation-positive non-small-cell lung cancer (NSCLC) is still an important fundamental problem. Ethacrynic acid (EA) has been proved as a dual inhibitor of GST and WNT, and the *α*, *β*-unsaturated-keto structure of it is similar to that of irreversible tyrosine kinase inhibitors (TKIs). However, these beneficial effects of EA combined with afatinib have never been reported in NSCLC. Therefore, the antitumor effects of afatinib combined with EA in EGFR L858R/T790M-mutated NSCLC cells and related mechanisms were analyzed. Our *in vitro* and *in vivo* results showed that EA has strong synergistic antitumor effects with afatinib in EGFR L858R/T790M-mutated NSCLC cells, but has no cytotoxic effects in NSCLC cells when used it alone, i.e., the cytotoxic effects of afatinib (IC30) plus EA (IC30) were stronger than the effects of afatinib (IC50) alone. Our functional studies found that the antitumor mechanisms of afatinib when combined with EA mainly occurred by inhibiting WNT/*β*-catenin pathway activation and suppression of the secretion of anti-inflammatory factors. These results revealed that combination of afatinib with EA derivatives not only provided a new therapeutic approach for EGFR/T790M-mutated NSCLC patients but also offered a new idea for developing new drugs or optimizing the dose of afatinib in clinical use in future antitumor therapy.

## 1. Introduction

Lung cancer is the most prevalent and lethal type of cancer worldwide, and approximately 80% of all these cases are non-small-cell lung cancer (NSCLC). It is estimated that 135,720 (72,500 men and 63,220 women) deaths occur due to this disease in the year 2020 based on the report of *Cancer Statistics* [1]. Unfortunately, most of the lung cancer patients (about 50%) are diagnosed at advanced stages and have metastatic cancer, missing the opportunity of surgical treatment [2]. Although epithelial growth factor receptor (EGFR) mutation-positive advanced/recurrence NSCLC can receive EGFR tyrosine kinase inhibitors (TKIs) as a promising targeted treatment; their 5-year relative survival rate is just 5% after treatment [1]. This meant that the current treatment strategies are not effective in suppressing the lung cancers. Thus, how to improve the therapeutic efficiency and prolong survival time is an urgent problem to be solved in lung cancer.

Afatinib is an irreversible, second-generation tyrosine kinase inhibitors (TKIs) and an effective first-line treatment strategy for patients with EGFR-mutant NSCLC [3]. Recent data from real-world studies and LUX-Lung 8 together revealed that afatinib has not only a good response rate and could prolong median progression-free survival (PFS) rate at 12 months but also benefit patients with rare or complex EGFR mutations and symptomatic brain metastases [4–7]. Moreover, a recent study showed that sequential treatment with afatinib and osimertinib in patients with EGFR-T790M mutant NSCLC demonstrated an overall median survival time of 27.6 months after treatment, 30.3 months in Del19-positive patients, and 46.7 months in Asians. Additionally, the 2-year overall survival (OS) rate is 78.9% [8]. These findings proved afatinib as a potent and highly selective drug for treating NSCLC in patients. Afatinib treatment is widely accepted due to its inhibition of epidermal growth factor receptors 1 (ErbB1; EGFR), 2 (ErbB2; HER2), and 4 (ErbB4; HER4) and certain EGFR mutants, including those caused by EGFR exon 19 deletion mutations or exon 21 (L858R) mutations. It is also associated with severe side effects in one-tenth of patients, and the most common side effects were diarrhea, paronychia, and fatigue [5, 9]. Additionally, similar to the first-generation TKIs, EGFR T790M mutation is regarded as the major mechanism of acquired resistance to afatinib [10]. Hence, it is essential to find new strategies to improve the therapeutic effects of afatinib and overcome acquired resistance or side effects.

Ethacrynic acid (EA) is a diuretic agent clinically and has been confirmed to act as a WNT and GST inhibitor. It has selective toxicity against chronic lymphocytic leukemia cells [11], multiple myeloma [12], and pancreatic cancer [13]. Recently, EA has been reported to have synergistic antitumor effects in breast cancer when combined with irreversible EGFR TKIs [14]. Moreover, one study revealed that *β*-catenin of the classical WNT pathway contributed to the development of lung tumors induced by EGFR-T790M mutations, and genetic deletion of *β*-catenin gene dramatically reduced lung tumor formation in EGFR-L858R-T790M transgenic mice [15]. These findings revealed EA as a WNT inhibitor and could help to resolve the problem of EGFR-TKI's acquired resistance. However, the antitumor effects of EA combined with afatinib in NSCLC have never been studied. Thus, we aimed to explore whether EA could enhance the antitumor effects of afatinib in NSCLC and reveal the relative mechanism.

## 2. Materials and Methods

### 2.1. Cell Lines and Cell Culture

The human NSCLC cell lines A549 and H1975 were purchased from the Cell Biology of Chinese Academy of Science (Shanghai, China). The cells were cultured in RPMI-1640 (Gibco, USA) supplemented with 10% fetal bovine serum (BIOIND, Israel), 100 *μ*g/ml streptomycin and 100 U/ml penicillin (Gibco, USA) at 37°C in a humidified 5% CO_2_. The cells were passaged every 2-3 days by 0.25% trypsin (Gibco) and not cultured for more than 3 months.

### 2.2. Cytotoxicity Assay

CCK8 assay was used to detect the drug cytotoxic effects. Briefly, the cells at a density of 5 × 10^3^/well were plated in a 96-well plate and incubated for 24 h followed by treatment with afatinib with or without EA for 48 h. Next, the cells were stained with CCK8 (Dojindo, Japan) for 2 h. The absorbance was then measured at 450 nm using a microplate reader (Thermo, USA).

### 2.3. Drugs and Reagents

Afatinib and EA were obtained from Melone Pharmaceutical Company (China) and Sigma (USA). These substances were diluted in DMSO and stocked at a concentration of 10 mm for afatinib and 100 mm for EA. These were diluted to five different concentrations to stimulate cells. The IC50 value was analyzed based on the data of cytotoxic effects after treatment for 48 hours at this time point. Additionally, the synergistic effect of the two drugs or the coefficient of drug interaction (CDI) was analyzed using the Calcusyn software [16]. CDI less than 0.7 (CDI < 1) indicates a significant synergistic effect; CDI = 1 represents that the two drugs have an additive effect; and CDI > 1 represents that the two drugs have antagonistic effects.

### 2.4. Cell Cycle and Apoptosis

The cells at a concentration of 2 × 10^5^/ml/well were plated in a 6-well plate and incubated overnight. The cells were treated with afatinib with EA or EA alone for 48 h. The cells were then harvested for the following analysis. The cells were fixed in 75% ethanol for overnight at 4°C and centrifugation followed by washing with cold PBS three times and treatment with 50 *μ*l of RNase A at a final concentration of 100 *μ*g/ml for 1 h at room temperature for cell cycle analysis. Propidium iodide staining buffer (PI, final concentration is 50 *μ*g/ml) (Shanghai Yuanmu Biological Technology Co. Ltd, China) was then added to each well until it reaches a final volume of 500 ml. The cell cycle was then analyzed by flow cytometry (Beckman Counter, USA). Cell apoptosis was detected using an apoptosis Kit (Becton-Dickinson, USA) according to the kit protocol. After treatment with afatinib and with/without EA for 48 h, the cells and the supernatant were collected, incubated with FITC Annexin V and PI for 30 min, and measured using FACS Calibur flow cytometer (Becton-Dickinson, USA).

### 2.5. Animal Study

Male BALB/C nude mice (4-5 weeks old, 16-20 g) were obtained from Guangxi Medical University (Nanning, China) and housed in Guangxi Medical University Laboratory Animal Center (Nanning, China). All animal experiments were conducted according to the Guangxi Medical College Animal Care Committee's ethical and animal experiment regulations. For tumor cell inoculation, A549 or H1975 cells (8 × 10^6^ cells were suspended in 100 *μ*l PBS) were injected subcutaneously into the left flank to produce subcutaneous tumors. The tumor-bearing mice, those that did not form tumors or the smallest tumors were removed, were randomly divided into four groups (six mice per group) when the tumor size reached to 150 to 200 mm^3^: (1) control group (100 *μ*l PBS); (2) afatinib group (25 mg/kg/daily); (3) EA group (20 mg/kg/daily); and (4) afatinib+EA group (25 mg/kg/daily + 20 mg/kg/daily) [14, 17]. All mice were treated with the above-mentioned drugs by intragastric administration for 3 weeks. Finally, the tumor size and body weight were measured according to the formula Tumor volume = 0.5 × length × width^2^.

### 2.6. RNA Extraction and Quantitative RT-PCR

Total RNA was extracted using trizol reagent (Invitrogen, Carlsbad, CA, USA) according to the manufacturer's instructions. cDNAs were synthesized using the ReverTra Ace qPCR RT kit (FSQ-101; Toyobo, Kagoshima, Japan). Real-time PCR analyses were performed with Thunderbird SYBR qPCR mix (QPS-201; Toyobo) on an MxPro Mx3000P Sequence Detection system (Stratagene, La Jolla, CA, USA). *β*-Catenin was used as an internal normalized reference, and fold changes were calculated by relative quantification (2^−ΔΔCt^). The primer sequences are shown in supplemental Table 1.

### 2.7. Western Blotting

EGFR, WNT7B, *β*-catenin, RET, and GAPDH were purchased from Cell Signaling Technology (CST). The cells were harvested and lysed with RIPA protein extraction reagent supplemented with protease inhibitor cocktail. The protein concentrations were measured using the BCA assay (Pierce, CA, USA). Equal amounts of extracts were loaded and separated by electrophoresis on 8-10% SDS-PAGE and transferred onto nitrocellulose membranes (Bio-Rad). The membranes were blocked for 1 h at room temperature in Tris-buffered saline/0.1% Tween 20 (TBST) containing 5% (wt/vol) nonfat milk and then incubated with primary antibodies in TBST containing 5% (wt/vol) nonfat milk or 5% (wt/vol) BSA at 4°C overnight. The membranes were then incubated with an appropriate secondary antibody coupled to horseradish peroxidase, and the proteins were detected by ECL Supersignal West Pico Chemiluminescence Kit (Thermo Fisher Scientific).

### 2.8. RNA Sequencing and Data Analysis

The H1975 NSCLC cells after treatment with different drugs for 24 h were sent to Yucebio Company (Shenzhen, China) and underwent RNA sequence analysis. Meanwhile, for differential gene expression analysis, Gene Ontology (GO) and Kyoto Encyclopedia of Genes and Genomes (KEGG) pathways were analyzed. Briefly, differential expression analysis was performed using the DESeq (V1.6.3) and EdgeR (V3.4.6) Bioconductor package. The data were adjusted using the Benjamini and Hochberg approach for controlling the false discovery rate. The *p* value was set to *p* < 0.05 to detect the differentially expressed genes (DEGs). KEGG pathway analysis was used to harvest the pathway clusters of molecular interaction and reaction networks in differentially regulated gene profiling. In the present study, significant pathways were identified as those with a fold change of ≥2 and *p* values of <0.05.

### 2.9. Statistical Analysis

Data were presented as means ± standard deviation (S.D.) of one representative experiment. Unless otherwise noted, statistically significant differences were analyzed by one-way analysis of variance (ANOVA) when there were more than two groups. All analyses were performed using GraphPad Prism 8. In all analyses, *p* < 0.05 was considered as statistical significance.

## 3. Results

### 3.1. The Validation of the Cytotoxic Effects of Afatinib and EA in NSCLC Cells

**Figure 1:**
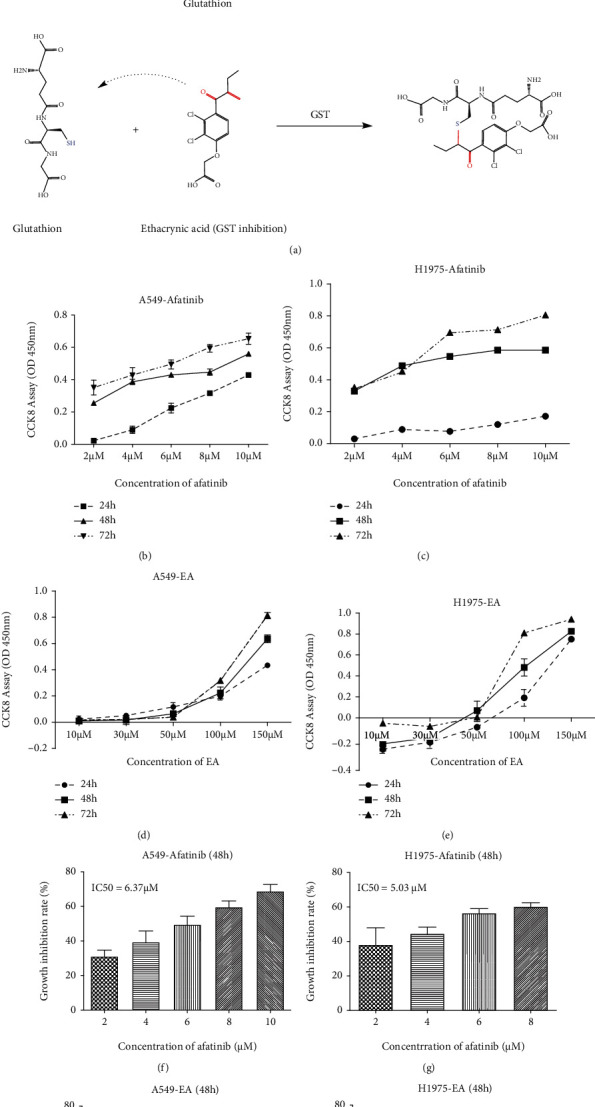
The validation of cytotoxic effects of afatinib and EA in NSCLC cells. (a) Interaction between afatinib or ethacrynic acid and glutathione. (b–e) The cell proliferation of A549 or H1975 cells after treatment with afatinib or EA at different time points. (f–i) IC50 value of afatinib or ethacrynic acid in different cells at 48 h. The IC50 value is the mean concentration of drug that reduced cell survival by 50%.

To assess the cytotoxic effects of afatinib or EA on NSCLC cell lines, A549 cells (EGFR^WT^) and H1975 cells (EGFR^L858R/T790M^ mutation) were used. As shown in Figures 1(b) and 1(c), afatinib significantly inhibited the growth of NSCLC cells (A549 and H1975), and this inhibition was increased correspondingly with increasing drug concentrations and time. However, low concentrations of EA promoted H1975 cell proliferation even though high concentration of EA exerted cytotoxic effects in A549 cells and H1975 cells (Figures 1(d) and 1(e)). When the IC50 value was calculated using the data at 48 h, the results showed that the IC50 of EA in A549 or H1975 reached the highest to 87.03 *μ*M or 99.54 *μ*M, respectively (Figures 1(h) and 1(i)), indicating that the mean EA had little effect on NSCLC cells. In contrast, the cytotoxic effects of afatinib with EA were more significant in EGFR-L858R-mutated H1975 cells (IC50 = 5.03 *μ*M) than that in EGFR-WT A549 cells (IC50 = 6.37 *μ*M). These findings suggested that L858R-EGFR-mutated NSCLC cells were more sensitive to afatinib than WT-EGFR cells.

### 3.2. EA Combined with Afatinib Had Synergistic Cytotoxic Effects on EGFR^L858R/T790M^-Mutated NSCLC Cells *In Vitro*

**Figure 2:**
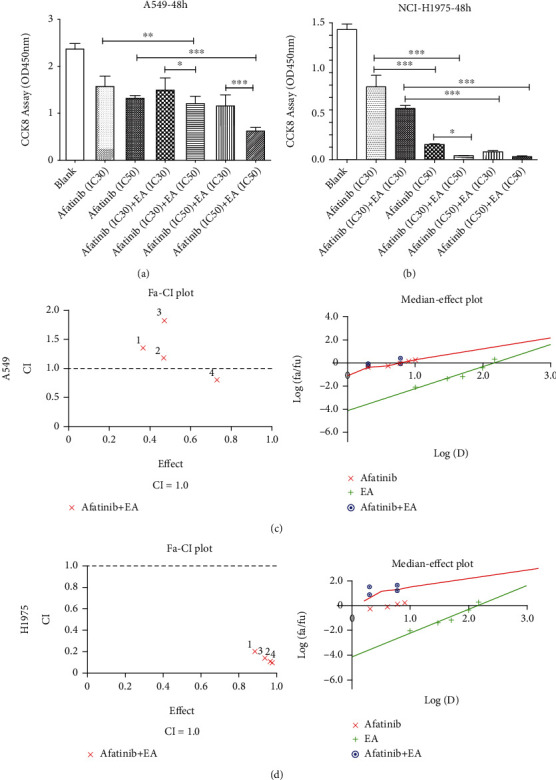
The effects of combination of EA and afatinib on A549 and H1975 cells. (a) The cytotoxic effects of afatinib combined with or without EA in A549 cells. (b) The cytotoxic effects of afatinib combined with or without EA in H1975 cells. The Calcusyn software was used to analyze *the* combination drug index (CDI) in A549 (c) or H1975 (d) cells. CI: coefficient index; Fa: the fraction affected by dose; Fu: The fraction unaffected; Fu = 1 − Fa. ^∗^*p* < 0.05; ^∗∗^*p* < 0.01; ^∗∗∗^*p* < 0.001.

To further explore whether EA combined with afatinib has synergistic antitumor effects in NSCLC cells as previously reported in breast cancer [14], a dosage of IC30~50 of these two drugs was combined. As shown in Figures 2(a) and 2(b), EA combined with afatinib significantly inhibited H1975 cell proliferation when compared with afatinib alone, while this combination effect was not so obvious in EGFR-WT A549 cells. Besides these, Calcusyn software was used to analyze the combination drug index (CDI) in different cells. As shown in Table 1 and Figures 2(c) and 2(d), regardless of which concentration of EA (IC30--75*μ*M or IC50--100*μ*M) combined with 2 *μ*M afatinib (IC30) or 6 *μ*M afatinib (IC50), the CDI of afatinib combined with EA in H1975 cell was less than 0.2. On contrary, the CDI of afatinib plus EA in A549 cells was larger than 0.8, and even larger than 1 at times, which meant that they had antagonistic effects. These findings indicated that EA plays a synergistic role and enhanced the cytotoxic effects of afatinib in EGFR-mutated NSCLC cells.

### 3.3. Combination Treatment with EA and Afatinib Enhanced Antitumor Effects In Vivo

**Figure 3:**
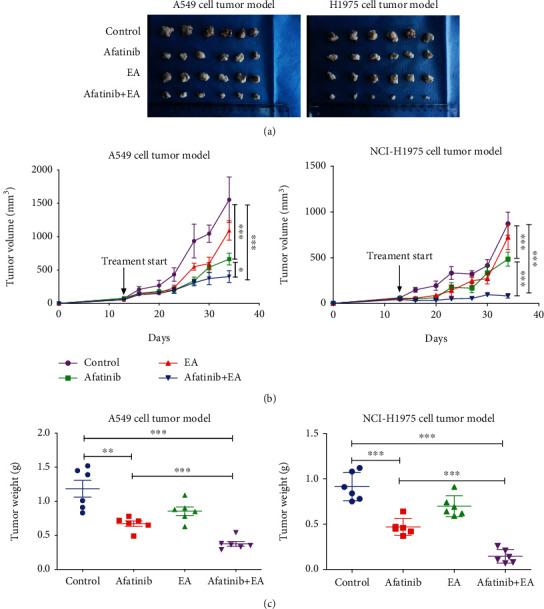
Combination of afatinib with EA suppressed tumor growth *in vivo*. (a) Image showing the method of tumor removal in a representative experiment. (b) The change in tumor volume of mice after treatment with different drugs. (c) Tumor weights of mice after treatment with different drugs. ^∗^*p* < 0.05; ^∗∗^*p* < 0.01; ^∗∗∗^*p* < 0.01.

To evaluate whether combined treatment with EA and afatinib had stronger antitumor effects *in vivo*, A549 and H1975 NSCLC cells were implanted subcutaneously into the back of syngeneic Balb/c mice. When the tumor diameter of these reached to 5 mm, the mice were treated by intragastric administration with afatinib (25 mg/kg) alone or together with EA (20 mg/kg) for 3 weeks. All the nude mice were put to death after anesthesia, the tumors were separated and weighed. The calculation formula of tumor inhibition rate is as follows: (TW_Control group_ − TW_experimental group_)/TW_Control group_ × 100%. The results as shown in Figure 3 and Table 2 revealed that the tumor rate in the combination group (84.12%) was significantly higher than that in the afatinib alone group (48.72%) in H1975-tumor model, while the tumor inhibition rate in the combination group (69.76%) was increased slightly when compared to that in the afatinib group (51.75%).

### 3.4. EA Enhanced the Antitumor Effects of Afatinib by Inhibiting Cell Cycle Progression and Inducing Cell Apoptosis in EGFR L858R/T790M-Mutated NSCLC Cells

**Figure 4:**
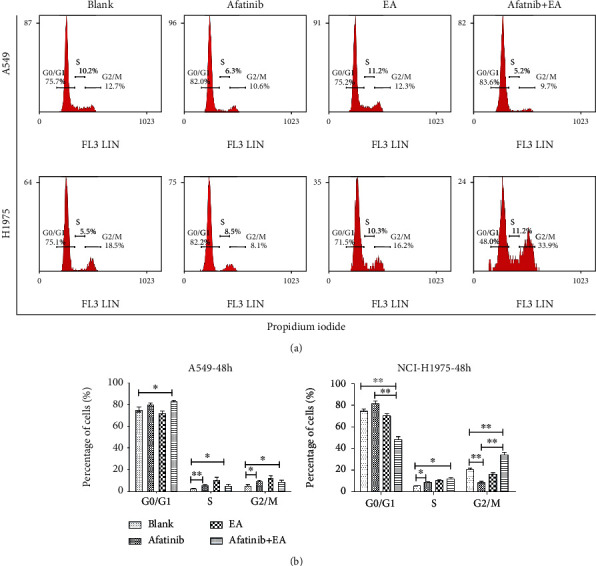
The effect of combination afatinib with EA on cell cycle. (a) A549 and H1975 cells were treated with afatinib and with or without EA for 48 hours after the cells were harvested and analyzed by FACS. (b) The calculated cell cycle distribution. Data are presented as means ± SD (*n* = 6) of a representative experiment. Similar results were obtained in three experiments. ^∗^*p* < 0.05; ^∗∗^*p* < 0.01; ^∗∗∗^*p* < 0.001.

**Figure 5:**
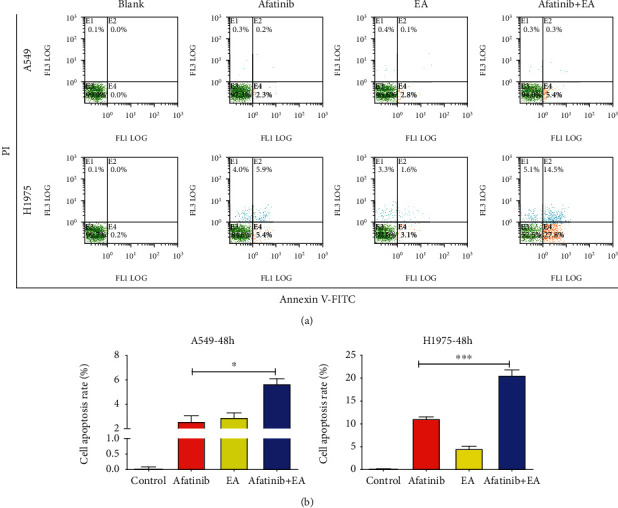
The effects of combination of afatinib with EA on cell apoptosis. (a) A549 and H1975 cells were treated with afatinib and with or without EA for 48 hours after the cells were harvested and analyzed by FACS. (b) The calculated cell cycle distribution. Data are shown as means ± SD (*n* = 6) of a representative experiment. Similar results were obtained in three experiments. ^∗^*p* < 0.05; ^∗∗^*p* < 0.01; ^∗∗∗^*p* < 0.001.

To investigate the effects of EA combined with afatinib on NSCLC cell function, a 30% ~50% inhibitory concentration of afatinib (6 *μ*M) and EA (75 *μ*M) was chosen for subsequent experiments. Thus, we examined if there were any changes in the cell cycle and apoptotic rate that are associated with tumor cell growth. Cell cycle analysis revealed that EA combined with afatinib significantly reduced the G0/G1 phase (afatinib vs. afatinib +EA: 83.6% vs. 48.0%) and blocked the cell cycle at G2/M phase, controlling the cell division process (afatinib vs. afatinib +EA: 9.7% vs. 33.9%) (Figure 4). Additionally, apoptosis results revealed that the apoptotic rate of H1975 in EA combined with afatinib group was significantly higher than that in the afatinib alone group, and the apoptotic rate of combination group and afatinib alone was about 20% and 10%, respectively (Figure 5). However, the cell cycle results and apoptotic rate in the combination group showed no significant difference when compared to afatinib alone group in A549 cells. These results were consistent with the results of tumor growth.

### 3.5. EA Enhanced the Antitumor Effects of Afatinib in NSCLC by Suppressing WNT/*β*-Catenin Pathway Activation

**Figure 6:**
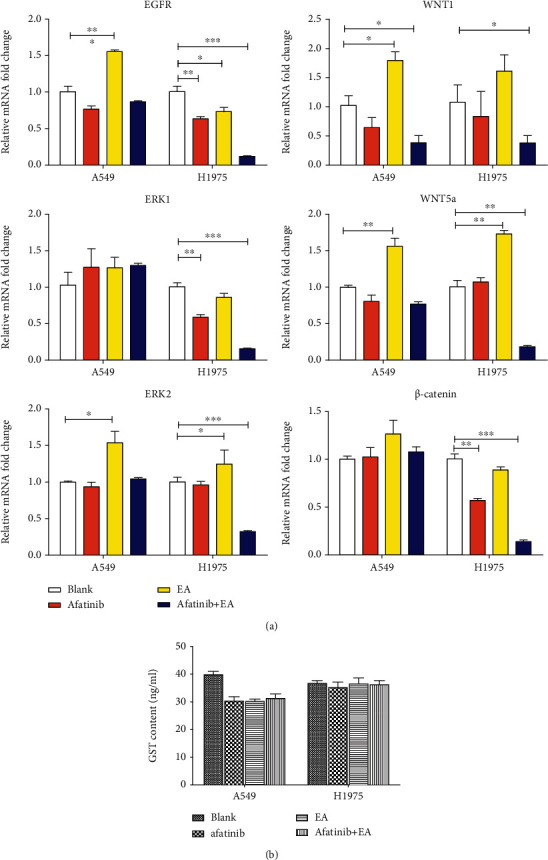
Combination with afatinib and EA inhibited EGFR pathway and WNT pathway. A549 and H1975 were analyzed by real-time qRT-PCR after stimulation with afatinib and with or without EA for 48 hours. (a) The impact on EGFR, ERK1, ERK2, WNT1, WNT5A, and *β*-catenin. (b) The change of GST protein in A549 or H1975 cells after treatment with afatinib and with or without EA. ^∗^*p* < 0.05; ^∗∗^*p* < 0.01; ^∗∗∗^*p* < 0.001.

Previous studies have reported that EA acts as a dual inhibitor of GST and WNT, and afatinib can inhibit EGFR family, and so we examined whether the combination of EA and afatinib has enhanced antitumor effects in NSCLC by suppressing EGFR and WNT signaling pathways. The WNT proteins were grouped as classical WNTs (WNT1) that activate the *β*-catenin-dependent (canonical) pathway and nonclassical WNTs (WNT5A) for inducing *β*-catenin-independent (noncanonical) signaling pathways [18]. The mRNA expression of EGFR and its downstream ERK1/2, WNT1, WNT5A, and *β*-catenin was detected in this study. As shown in Figure 6(a), compared to afatinib alone group, the combination of EA and afatinib has significantly cosuppressed the mRNA expression of EGFR/ERK1/2 and WNT1/*β*-catenin and WNT5A in H1975 cells, while there was no significant difference in the combination group when compared with the afatinib alone group in A549 cells. In addition, GST levels were also detected in this study using ELISA, but no matter what type of NSCLC cells, afatinib alone or combined treatment showed no change in the expression of GST protein (Figure 6(b)). Therefore, GST was hypothesized to be mainly synthesized and secreted by hepatocytes and its protein level remained very low in NSCLC cells, making it difficult to detect significant changes in protein expression. The above results indicated that the combination treatment was more effective in EGFR-L858R/T790M-mutated NSCLC cells than in EGFR-WT NSCLC. Hence, further exploration of the real underlying mechanism of combined treatment in H1975 cells using RNA sequencing is warranted.

**Figure 7:**
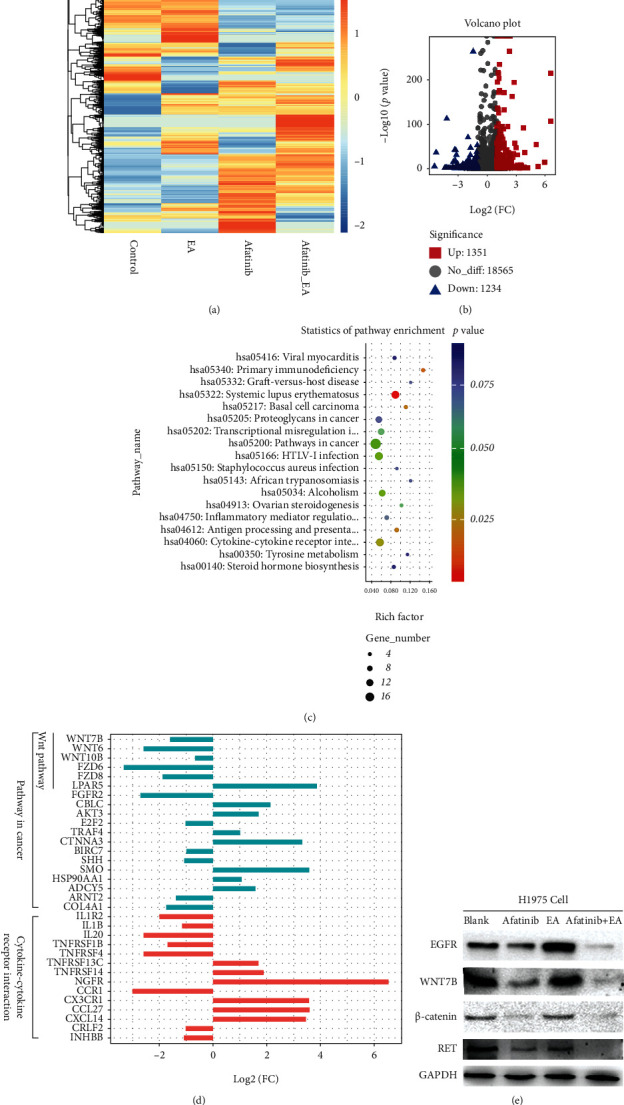
The mechanism of EA combined with afatinib had synergistic cytotoxic effects on H1975 cells. (a) The heat map showed gene expression of H1975 from RNA-seq after treatment with afatinib and with or without EA for 24 h. (b) Volcano plot showed the expression of differential genes in afatinib combined EA vs. afatinib alone. (c) Statistics of pathway enrichment on differential expressed genes were analyzed by KEGG (afatinib+EA vs. afatinib). (d) Relative expression of genes in the cancer and cytokine-cytokine receptor interaction pathways. (e) The change of protein expression in H1975 after treatment with afatinib and with or without EA were examined by western blotting.

As shown in Figure 7(a), the heat map showed gene expression changes, in which the combination group reversed most of the gene expression changes when compared with the afatinib alone group. Similar significant gene profile changes were obtained in H1975 cells. Next, the DEGs between afatinib and afatinib combined with EA were focused on, and a volcano plot was used to show the DEGs with a fold change of ≥2 and *p* value of ≤0.05. As shown in Figure 7(b), there were 1351 upregulated genes and 1234 downregulated genes. David 6.8 was used as a functional annotation tool to enrich these DEGs and the results of Pathway Enrichment. The top 3 enrichment pathways were shown in cancer (19 genes), cytokine-cytokine receptor interaction (14 genes), and HTLV-I infection (14 genes), respectively (*p* < 0.05), (Figure 7(c)). Beyond this, the pathway in cancer revealed that most of the genes are relative to WNT pathway (WNT7B, WNT6, WNT10B, FZD6, FZD8, and LPAR5), and among these, WNT7B, WNT10B, FZD6, FZD8, and LPAR5 belonged to the classical WNT pathway. As cytokines have paracrine action and play an important role in the tumor microenvironment, the cytokine-cytokine receptor pathway was analyzed and found that many anti-inflammatory factors (IL1R2, IL1B, and IL20) were decreased. The above-mentioned DEGs in the combination group when compared to afatinib alone group were displayed in Figure 7(d). Finally, the expression of some proteins was validated by western blotting and found that the protein expression of EGFR, WNT7B, and RET, *β*-catenin in the combination treatment group was significantly decreased when compared to that in afatinib. RET gene is a new target closely related to the pathogenesis of NSCLC. It mainly induces oncoprotein production through KIF5B-RET, CCDC6RET, NCOA4RET, and TTlM33 genes and activates signal transduction pathways similar to ALK gene mutations and carcinogenesis. Mutations and fusions occur and are highly expressed in tumor tissues, thereby inducing NSCLC. As expected, the RET protein expression was significantly inhibited in the combination group than that in afatinib. Our findings demonstrated that combination with EA and afatinib enhanced the antitumor effects of afatinib and overcame T790M acquired resistance by suppressing WNT/*β*-catenin signaling pathway activation in NSCLC.

**Figure 8:**
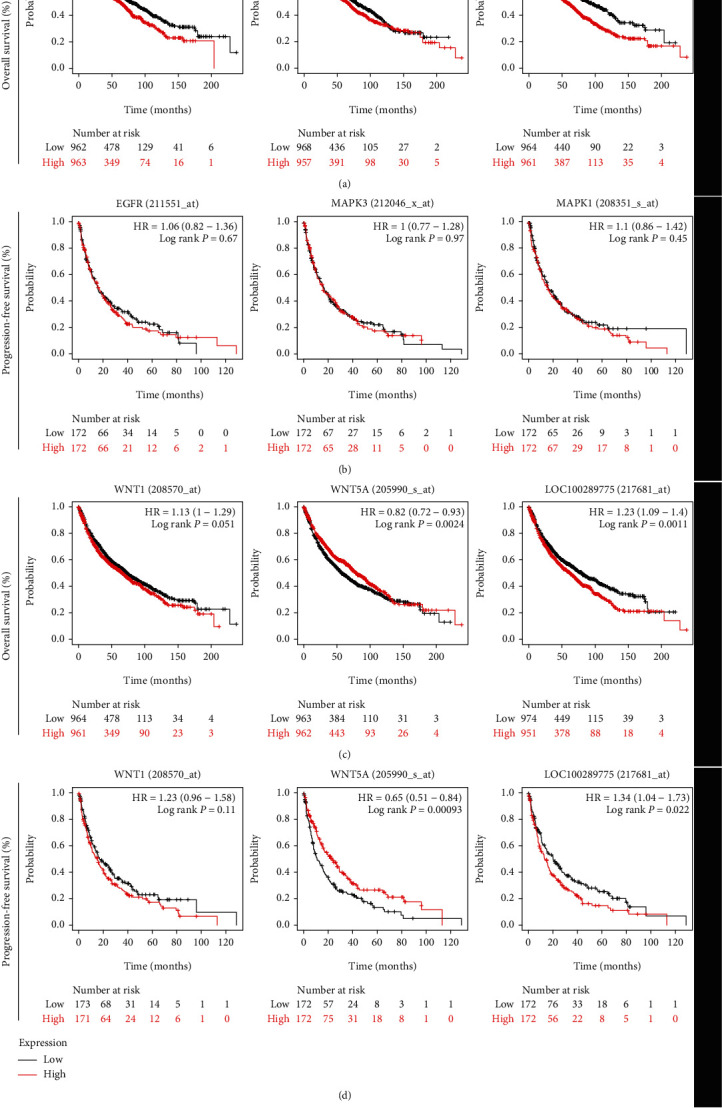
The effect of EGFR/ERK1/ERK2 OR WNT1/WNT5A/WNT7B gene expression on the OS and PFS of lung cancer patients by Kaplan-Meier curve and Log-rank test.

Furthermore, whether EGFR/ERK1/ERK2 or WNT1/WNT5A/WNT7B gene expression was potentially associated with the OS of lung cancer patients was assessed by Kaplan-Meier curve and Log-rank test. Kaplan-Meier curve plotter online tool (http://kmplot.com) included 1972 lung cancer patients for OS and 344 lung cancer patients for progression-free survival (PFS) to analyze their correlation. However, except for WNT1, the WNT5A and WNT7B gene expressions showed significant positive correlation with OS (Figure 8(c)) and PFS (Figure 8(d)). These data suggested that WNT signaling pathway activation might contribute to lung cancer progression or EGFR TKIs resistance. Hence, these findings demonstrated that combining EA and afatinib enhanced the antitumor effects of afatinib and overcame T790M acquired resistance by suppressing WNT/*β*-catenin pathway activation in NSCLC patients.

## 4. Discussion

Acquired resistance is an inevitable question for the long-term use of TKIs, and so how to overcome resistance and prolong the duration of drug application is not only a hot topic in the current research but also an urgent problem to be resolved. Hence, in this study, a combination treatment with afatinib and EA was used in NSCLC and found that EA has synergistic effects on the antitumor activity of afatinib in EGFR L858R/T790M-mutated NSCLC cells.

Afatinib is a good and irreversible EGFR TKI, and recently, many clinical trials have proved that it can effectively prolong the median PFS and OS time in NSCLC patients when compared to the first-generation EGFR TKIs [19]. However, severe side effects and newer mutations induced acquired resistance, limiting its use clinically, and so some patients who acquired resistance to the first generation TKIs directly jumped to the third generation EGFR TKI treatment like osimertinib [20, 21]. Even though osimertinib has been approved for the treatment of EGFR-T790M mutant NSCLC patients, it is associated with drug resistance [22]. Thus, how to prolong the duration of second generation EGFR TKIs before the occurrence of T790M mutation or overcome the acquired resistance assists in improving the cure rate in patients and is the problem to be solved in our study. Afatinib combined with EA in NSCLC was studied due to two main reasons: one is a paper which revealed that *β*-catenin of the classical WNT signaling pathway contributed to lung tumor development induced by EGFR-T790M mutations, and genetic deletion of *β*-catenin gene dramatically reduced lung tumor formation in EGFR-L858R-T790M transgenic mice [15], and the other one is EA as a glutathione S-transferase P1-1(GSTP1-1) and WNT inhibitor can improve the antitumor effects of irreversible EGFR TKIs in breast cancer [14]. Thus, we inferred that EA combined with afatinib could improve the antitumor effects of afatinib in acquiring resistance in NSCLC. Besides these, there are two main types of EGFR-TKIs resistance: primary resistance and acquired resistance [23]. For primary resistance, it is said that approximately 30% EGFR-mutated NSCLC patients develop resistance at the beginning of EGFR-TKI treatment due to K-Ras mutation and PTEN deletion [24–27]. For acquired resistance, EGFR-T790M mutation, MET gene amplification, and HGF overexpression can cause this [28]. In the study, it was found that IL1R2 is more likely to act as a carcinogen in tumors, and it is only lowly expressed in a few tumors. IL-1B has strong proinflammatory activity and activates related signal pathways after binding to receptors on target cells. Such as MAPK, IL-1 signaling pathway, and STAT3 signaling pathway, which induce tumor cell proliferation, migration, invasion, and metastasis [29, 30], IL-20 can activate the STAT signaling pathway as an effective angiogenesis, chemotaxis, and proinflammatory cytokine, which is related to chronic inflammatory diseases such as psoriasis, rheumatoid arthritis, osteoarthritis, cancer, and liver fibrosis [31]. A study showed that a single nucleotide polymorphism (SNP) of IL1R2 was found during the occurrence and development of NSCLC [32]. But their specific mechanisms affecting the process of lung cancer have not been reported in the literature. Among them, T790M mutation is considered the most important factor for secondary resistance to EGFR-TKIs, accounting for 50% of patients after EGFR-TKIs treatment [33]. Thus, A549 (EGFR wild-type and K-ras mutations) and H1975 (EGFR L858R and T790M mutations) NSCLC cells were chosen as research objects to better evaluate the antitumor effects of the combination of EA and afatinib [34]. Finally, our results showed that EA has no cytotoxic effects on NSCLC cells, and its IC50 value in A549 or H1975 cells reached to 87.03 *μ*M or 99.54 *μ*M, respectively. This high concentration does not meet the sensitivity and specificity requirements of drug development, and these results were not similar to those in leukemia [11, 35]. However, unexpectedly, regardless of whether 2 *μ*m (IC30) or 6 *μ*m (IC50) afatinib combined with 75 *μ*m (IC30) EA was used, the antitumor effects of these combinations were stronger than that of the same dose of afatinib in H1975 cells both *in vitro* and *in vivo*, and their combination drug index (CDI) was less than 0.2. Conversely, the combination has little effect on primary drug resistance in A549 cells, meaning that EA really has a synergistic effect on the antitumor effects of afatinib in EGFR-T790M-mutated NSCLC.

For the mechanism regarding the combination of EFGR-T790M-mutated NSCLC, RNAseq was used to comprehensively analyze. The data of the transcriptome as shown in the heat map revealed that the combination with afatinib and EA reversed most part of gene expression. Moreover, enriched and clustered analysis was performed for DEGs (log2 fold change > ∣1∣, *p* < 0.05) and found that the pathway in cancer was significantly enriched. Among these genes, WNT7B, WNT10B, FZD6, FZD8, and LPAR5 are classical WNT signaling pathway genes, and most of them were significantly suppressed in the combination group. These results were similar to the study conducted by Nakayama's group [15], in which EA can enhance the antitumor effects of afatinib in NSCLC by suppressing the classical WNT signaling pathway activation. However, no changes in GST were detected in this study. A meta-analysis in 2018 reported that glutathione S-transferase gene polymorphism (GST-PI) gene mRNA was high in NSCLC and was involved in the pathogenesis and prognosis of NSCLC [36, 37]. In contrast, a study reported that the levels of GSH were low in EGFR-T790M NSCLC and increased GSH expression in acquired NSCLC cells resensitized by the EGFR TKIs [38]. Regarding these, it is hypothesized that liver cytochrome P450 enzymes, glutathione, and other drug metabolism-related enzymes are mainly synthesized and secreted by the liver, and only *in vivo* experiments can offer reliable results for the detection of the effects of these enzymes on EGFR TKIs. However, there are some deficiencies that still require improvement. For example, it is still a question as to which targets of WNT signaling pathway can truly reverse or overcome drug resistance? How do the WNT signaling pathway and EGFR-related signaling pathways interact in NSCLC. More animal experiments and molecular experiments should be carried out in the future.

In conclusion, our results demonstrated that EA has synergistic effects in enhancing the antitumor effects of afatinib in EGFR-T790M-mutated NSCLC both *in vitro* and *in vivo* by suppressing WNT/*β*-catenin pathway. These studies provide strong evidence and experimental basis to overcome the resistance of afatinib and the development of more effective strategies for clinical application in the future.

## Figures and Tables

**Table 1:** The coefficient index (CI) of afatinib combined with EA in NSCLC cells.

Drugs	A549 cell		H1975 cell	
	Fa	CI	Fa	CI
Combination 1: Afatinib (2 *μ*M) + EA (75 *μ*M)	0.366151	1.362	0.883641	0.200
Combination 2: Afatinib (2 *μ*M) + EA (100 *μ*M)	0.465099	1.182	0.969724	0.114
Combination 3: Afatinib (6 *μ*M) + EA (75 *μ*M)	0.471219	1.830	0.93933	0.146
Combination 4: Afatinib (6 *μ*M) + EA (100 *μ*M)	0.730376	0.812	0.97684	0.101

CI: coefficient index; Fa: the fraction affected by dose; Fu: the unaffected fraction; Fu = 1 − Fa.

**Table 2:** The antitumor effects of EA combined with afatinib *in vivo* (*n* = 6).

Group	A549		H1975	
	Tumor weight (mg)	Inhibition rate (%)	Tumor weight (mg)	Inhibition rate (%)
Control	1.18 ± 0.30	0	0.91 ± 0.16	0
Afatinib	0.57 ± 0.11	51.75 ± 9.17	0.47 ± 0.09	48.72 ± 10.02
EA	0.83 ± 0.12	29.25 ± 10.37	0.70 ± 0.12	23.54 ± 12.76
Afatinib+EA	0.36 ± 0.05	69.76 ± 4.25	0.15 ± 0.08	84.12 ± 8.27
*F* value	18.345		50.192	
*p* value	0.000		0.000	

## Data Availability

The data used to support the findings of this study is included within the article, and the data are available from the corresponding author request.

## Supplementary Materials

Supplementary 1 Supplementary Materials 1: pathways to cancer new.

Supplementary 2 Supplementary Materials 2: pathways of cytokines.

Supplementary 3 Supplemental Table 1: primer sequences for quantitative RT-PCR.
