# Supplementary figures and images for: Ethacrynic Acid Enhances the Antitumor Effects of Afatinib in EGFR/T790M-Mutated NSCLC by Inhibiting WNT/Beta-Catenin Pathway Activation
Source: Dis Markers. 2021 Apr 27;2021:5530673. doi: 10.1155/2021/5530673 (PMC8168479; doi:10.1155/2021/5530673)

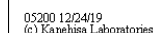

**List genes** are shown in red

Supplement: Supplementary 1 — Supplementary Materials 1: pathways to cancer new. [file 5530673.f1.pdf]
