# Supplementary material for: Ethacrynic Acid Enhances the Antitumor Effects of Afatinib in EGFR/T790M-Mutated NSCLC by Inhibiting WNT/Beta-Catenin Pathway Activation
Source: Dis Markers. 2021 Apr 27;2021:5530673. doi: 10.1155/2021/5530673 (PMC8168479; doi:10.1155/2021/5530673)
Supplement: Supplementary 2 — Supplementary Materials 2: pathways of cytokines. [file 5530673.f2.pdf]

Pathway:Cytokine-cytokine receptor interaction

Pathway information generated by KEGG. Stop Blinking

Help

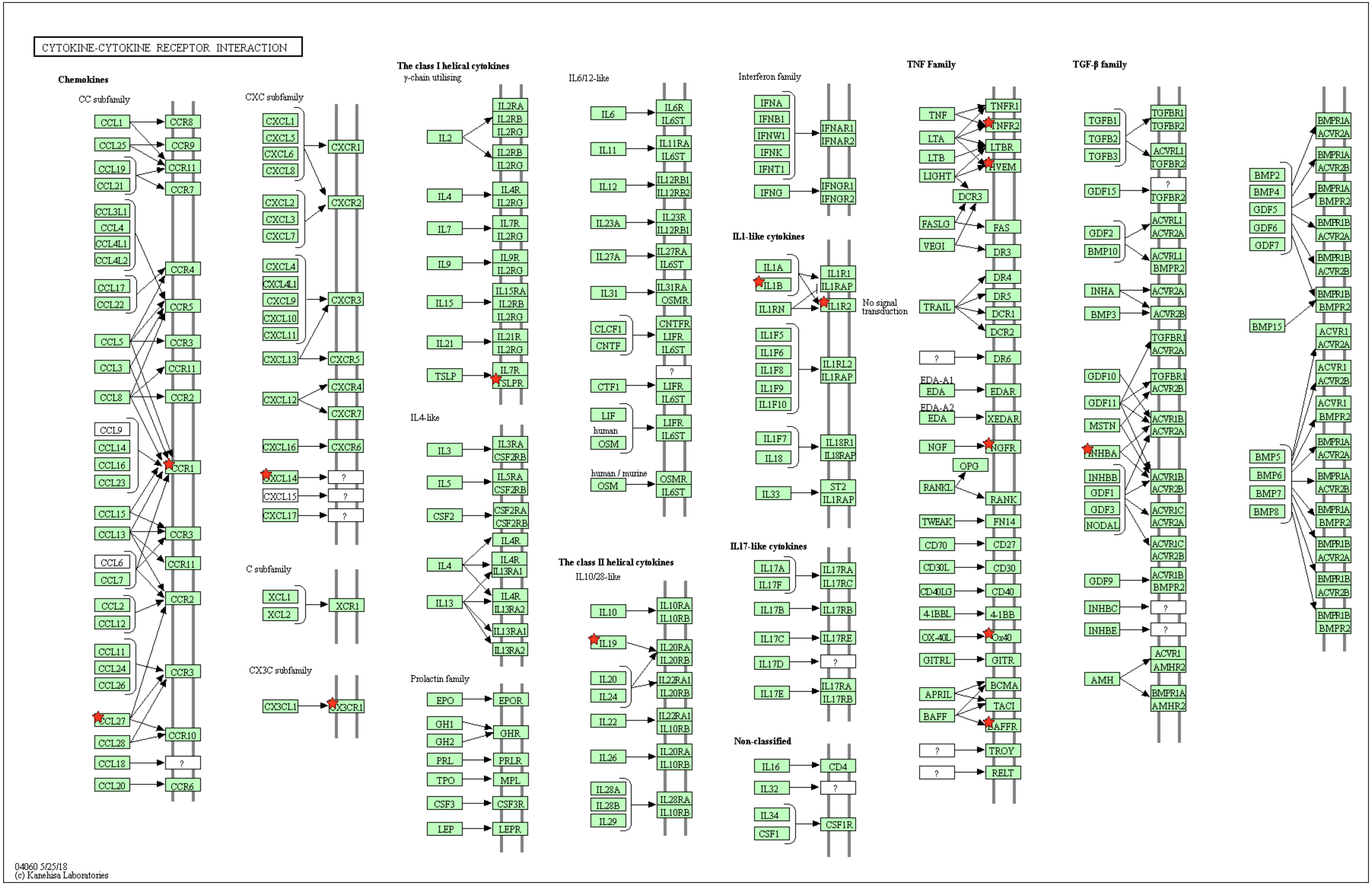

List genes are shown in red

DAVID Gene Name
C-C motif chemokine ligand 1(CCL1)
C-C motif chemokine ligand 11(CCL11)
C-C motif chemokine ligand 13(CCL13)
