# Supplementary material for: Ethacrynic Acid Enhances the Antitumor Effects of Afatinib in EGFR/T790M-Mutated NSCLC by Inhibiting WNT/Beta-Catenin Pathway Activation
Source: Dis Markers. 2021 Apr 27;2021:5530673. doi: 10.1155/2021/5530673 (PMC8168479; doi:10.1155/2021/5530673)
Supplement: Supplementary 3 — Supplemental Table 1: primer sequences for quantitative RT-PCR. [file 5530673.f3.docx]

Supplemental Table 1. Primer sequences for quantitative RT-PCR

| Gene | Primer Sequence |
| --- | --- |
| EGFR | F:5′-ATGAGATGGAGGAAGACGG -3′  R:5′-CGGCAGGATGTGGAGAT-3′ |
| WNT1 | F:5′-CAACCGAGGCTGTCGAGAAA-3′  R: 5′-GGCCGAAGTCAATGTTGTCG-3′ |
| ERK1 | F: 5′-GCTGACCCTGAGCACGACCA-3′ |
| ERK2 | R: 5′-CTCGTTCATCTGTCGGATCA-3′  F: 5′-CCCTTTGAGCACCAGACCTA-3′  R: 5′-GTGTTGAGCAGCAGGTTGGA-3′ |
| WNT5a | F: 5'‑ATGCAGTACATTGGAGAAGGTG‑3'  R:5'‑CGTCTCTCGGCTGCCTATTT‑3' |
| β-action | F: 5′-GAGACCTTCAACACCCCAGC-3′  R: 5′-ATGTCACGCACGATTTCCC-3′ |
